# Editorial: *eNeuro* Offers a Unique Interactive Experience to Reviewer Training

**DOI:** 10.1523/ENEURO.0157-17.2017

**Published:** 2017-05-24

**Authors:** Christophe Bernard

**Affiliations:** Editor-in-Chief

Dear friends and colleagues,

As you know, scientific research stands on three pillars: the observations and conclusions we generate in the lab, their validation by peer reviewing, and their publication. If one pillar starts to wobble, the science suffers. The quality of the data we produce is our sole responsibility. Everyone agrees that publishing one’s results can be a painful process, leading to the dissatisfaction of many authors. SfN journals along with other nonprofit journals are trying to improve the publication process to improve the experience for scientists. However, the problems of peer review remain poorly addressed. Everybody complains about the review process, myself included. We often feel mistreated by reviewers or editors, even if the latter are active scientists, as is the case for most nonprofit journals.

I believe that the core of the problem lies in the fact that we are rarely trained to review papers. As a consequence, our first exposure to the review process is passive, i.e., when we receive the reviews of the paper we have submitted. This becomes our “training” session, and later, we tend to reproduce it (perhaps subconsciously) when we review a paper for the first time. Knowing how best to review a paper requires training, and such training should happen as soon as possible during one’s career.

To aid this effort, SfN now offers regular peer review training sessions via webinar for SfN members on the Neuronline platform. The originality of our approach is to use real world examples. We select a paper that has been published by *eNeuro*. After registering, trainees can download the first submitted version of the paper and work on it using general guidelines we provide. During the webinar, we disclose the comments of the actual reviewers, along with the dialogue established between the Reviewing Editor and the reviewers, until they reached a consensus. You can then compare your own review with the synthesis of the reviews that was sent to the authors.

Two webinars are already available, and more are in preparation. Why do we need several different webinars? Interestingly, the way peer review is conducted appears to be specific to each neuroscience subfield. Modeling papers constitute a prototypical example. For example, the methods are rarely questioned in a modeling paper. After all, these are mathematical equations, and they can be either right or wrong. However, a model is usually made to provide a conceptual framework to understand a phenomenon. This often requires simplifying the system under scrutiny, which implies justifying what was kept and what was left out. In addition, there may be very different ways to model a phenomenon. Many modelers only like their own models. As a result, modelers tend to be the most critical reviewers of authors using a different approach. Therefore, computational neuroscience is a field where training sessions on how to review a modeling paper are very important. Our second webinar in this series focused on how to peer review a modeling paper, while our first webinar provided a more general introduction on how to peer review a manuscript.

We are now planning other webinars in other areas of neuroscience, since we believe that tailoring is in order. If you wish for a specific topic area to be covered, please do not hesitate to contact me at eneuroeditor@sfn.org.

Cheers,

